# A High-Frequency-Compatible Miniaturized Bandpass Filter with Air-Bridge Structures Using GaAs-Based Integrated Passive Device Technology

**DOI:** 10.3390/mi9090463

**Published:** 2018-09-13

**Authors:** Zhi-Ji Wang, Eun-Seong Kim, Jun-Ge Liang, Tian Qiang, Nam-Young Kim

**Affiliations:** 1 Radio Frequency Integrated Circuit (RFIC) Center, Kwangwoon University (01897), 20 Gwangwun-ro, Nowon-ku, Seoul 139-701, Korea; zhiji-wang@hotmail.com (Z.-J.W.); esk@kw.ac.kr (E.-S.K.); liangjun1991@hotmail.com (J.-G.L.); qtknight@hotmail.com (T.Q.); 2 Harbin Institute of Technology, School of Information and Engineering, Harbin 15001, China

**Keywords:** air-bridge structure, bandpass filter, capacitor, gallium arsenide, integrated passive device technology, intertwined inductor

## Abstract

This paper reports on the use of gallium arsenide-based integrated passive device technology for the implementation of a miniaturized bandpass filter that incorporates an intertwined circle-shaped spiral inductor and an integrated center-located capacitor. Air-bridge structures were introduced to the outer inductor and inner capacitor for the purpose of space-saving, thereby yielding a filter with an overall chip area of 1178 μm × 970 μm. Thus, not only is the chip area minimized, but the magnitude of return loss is also improved as a result of selective variation of bridge capacitance. The proposed device possesses a single passband with a central frequency of 1.71 GHz (return loss: 32.1 dB), and a wide fractional bandwidth (FBW) of 66.63% (insertion loss: 0.50 dB). One transmission zero with an amplitude of 43.42 dB was obtained on the right side of the passband at 4.48 GHz. Owing to its miniaturized chip size, wide *FBW*, good out-band suppression, and ability to yield high-quality signals, the fabricated bandpass filter can be implemented in various L-band applications such as mobile services, satellite navigation, telecommunications, and aircraft surveillance.

## 1. Introduction

With the rapid development of modern wireless and telecommunication systems, microwave filters have come to play an important role in various radio frequency (RF)/microwave applications because they allow frequency selection. Among the various types of microwave filters, bandpass filters (BPFs) have been extensively studied as a key building block in the design of RF/microwave integrated circuits and systems. However, emerging applications continue to challenge BPF efficacy with increasingly rigorous demands for lower cost, smaller size, higher performance, etc. [1,2,3].

To satisfy the increasingly stringent requirements of RF and microwave systems, a wide range of materials and techniques that can be used to modify existing lumped passive components have been incorporated into recently developed fabrication processes [4]. Recent advances in novel fabrication techniques, including the implementation of low-temperature co-fired ceramics (LTCC), high-temperature superconductor (HTS), micro-electromechanical system (MEMS), monolithic microwave integrated circuit (MMIC), and micro-fabrication techniques, have accelerated the rapid development of filters for various RF/microwave applications [5]. With LTCC technology, a variety of passive components such as strip lines, filters, antennas, and resonators, which are manufactured by using inexpensive yet highly conductive metals with low electrical resistance and low conductor loss at high frequencies, can be employed. However, this technology has two major disadvantages: (1) Post-firing ceramic shrinkage limits the size of the board to be processed, and (2) modules that require heat dissipation must be equipped with a heat sink that is applied after heating [6]. High-temperature superconductors are attractive because of their very low surface resistance in comparison to even the most conductive normal metals. Furthermore, their low loss properties allow the microwave passive components to be fabricated with a far more compact geometry than that of conventional materials. Despite its well-known advantages, the high cost and increased system complexity during low-temperature operation prevent HTS technology from being widely applied [7]. Micro-electromechanical system processing enables the batch fabrication of miniature structures that range in size from a few micrometers to a few millimeters; this technique facilitates cost, size, and weight reductions. Furthermore, the ability to seamlessly integrate mechanical components into electronics at the same wafer level is one of the most prominent properties of MEMS technology. However, MEMS components have a considerably short life cycle in addition to undefined power handling capability and an unstable ultimate dynamic range [8]. Alternatively, gallium arsenide and other advanced III–V materials allow MMICs to be applied in the millimeter wavelength region with increased functional capability, improved system reliability, and reduced weight, volume, and cost [9]. The major drawback of this technology is that the circuits can yield performance results for certain parameters that are worse than those observed from the same devices made with separate components [10].

Recently, some groups are researching the implementation of BPFs with emerging technologies such as complementary metal-oxide-semiconductor (CMOS) and stimulated Brillouin scattering (SBS). With regard to low-cost mass production, high integration capability, and lower power consumption, CMOS has proven to be a promising fabrication technique. However, this technology causes serious issues in the realization of high-quality passive components due to losses, especially at high frequencies [11,12,13]. Stimulated Brillouin scattering-based microwave photonics filtering has attracted significant interest over classical microwave techniques for its low propagation loss, ultra-broad transmission bandwidth, light-weight, and small-footprint. The bandwidth of the SBS-based filter can be adjusted by tailoring the pump profile and therefore provides the device with wide bandwidth tunability (MHz–GHz). Apart from the perfection, the SBS-based device is hard to be monolithically integrated with other on-chip photodetectors and electro-optic modulators [14,15,16]. In general, lumped elements have a lower Q value (for CMOS, Q factor is less than 15) [17,18]; to some extent, this problem can be mitigated by implementing integrated passive device (IPD) technology (Q factor >30), as is described in our previous reports [19,20,21,22]. Integrated passive device technology employs a newly developed fabrication technique that achieves the desired trade-off for package integration systems and affords complete module solutions; thus, overall improved system performance can be achieved through integration with other functionalities. This technique facilitates the design of a simplified and compact passive module with excellent parametric control capability and higher performance as compared to standard discrete systems owing to the reduced parasitic effect.

In this paper, we describe the design and implementation of a miniaturized bandpass filter with air-bridge structures that is fabricated on a gallium arsenide substrate by using IPD technology and is purposed for RF/microwave applications. An equivalent circuit was constructed for the model in consideration of the second-order parasitic effect that is generated under the condition of high frequency. Additionally, the current density was simulated at four selected frequency points by using a three-dimensional (3D) electromagnetic (EM) current simulator. Then, the influence of the inner radius of the BPF was experimentally investigated from the perspective of the S-parameter and lumped parameters.

## 2. Materials and Methods

### 2.1. Equivalent Circuit

**Figure 1:**
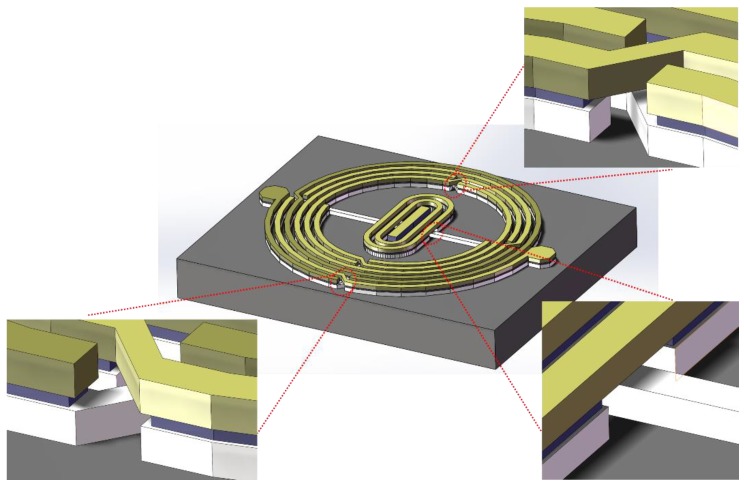
Three-dimensional layout of the proposed bandpass filter (BPF), showing the three laminated layers (bottom to top: Bond layer, Text layer, Leads layer).

**Figure 2:**
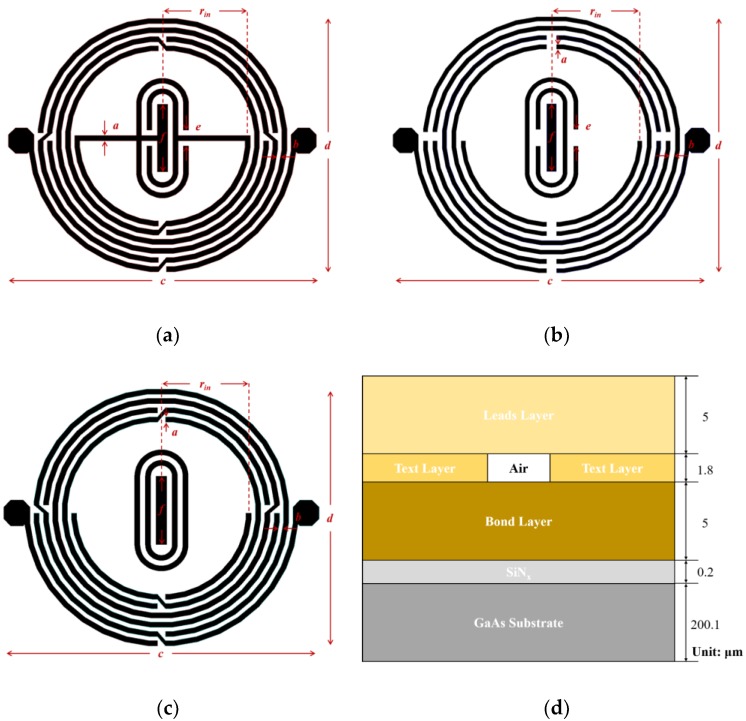
Configuration of the (**a**) Bond layer, (**b**) Text layer, and (**c**) Leads layer, and (**d**) a cross-sectional view of the fabricated integrated passive device (IPD) chip.

As illustrated in Figure 1, a quasi-interdigital capacitor was fabricated between the two divisions of a circle-shaped spiral inductor to build up a compact RF resonator. The proposed BPF was constructed with three copper layers, which, from bottom to top, are the Bond layer, Text layer, and Leads layer, having thicknesses of 5 μm, 1.8 μm, and 5 μm, respectively. Figure 2a–c illustrates the configurations of each of these three layers; their geometric parameters are respectively listed in Table 1, Table 2 and Table 3. A schematic diagram showing the cross-sectional view of the fabricated chip for the IPD is presented in Figure 2d.

The proposed BPF can be simplified as an *LC* resonant circuit, where an inductor (*L*) and a capacitor (*C*) are connected and have a resonant frequency, which is determined by the equation below:

(1) $$f_{0}=\frac{1}{2\pi \sqrt{LC}}$$

where *L* and *C* denote the inductance and capacitance, respectively. Moreover, the transmission zero can be derived using:

(2) $$w_{0}=\omega_{\mathrm{mid}}\frac{\Omega_{c}\times FBW+\sqrt{{\left(\Omega_{c}\times FBW\right)}^{2}+4}}{2}$$

where $\omega_{\mathrm{mid}}$ is the midband frequency, $\Omega_{c}$ is the passband cut-off frequency of the higher frequency area, and FBW is the fractional bandwidth, which can be calculated using:

(3) $$FBW=\frac{\omega_{2}-\omega_{1}}{\omega_{0}}$$

and

(4) $$\omega_{0}=\sqrt{\omega_{1}\omega_{2}}$$

where $\omega_{1}$ and $\omega_{2}$ indicate the passband-edge angular frequency, and $\omega_{0}$ denotes the center frequency.

**Figure 3:**
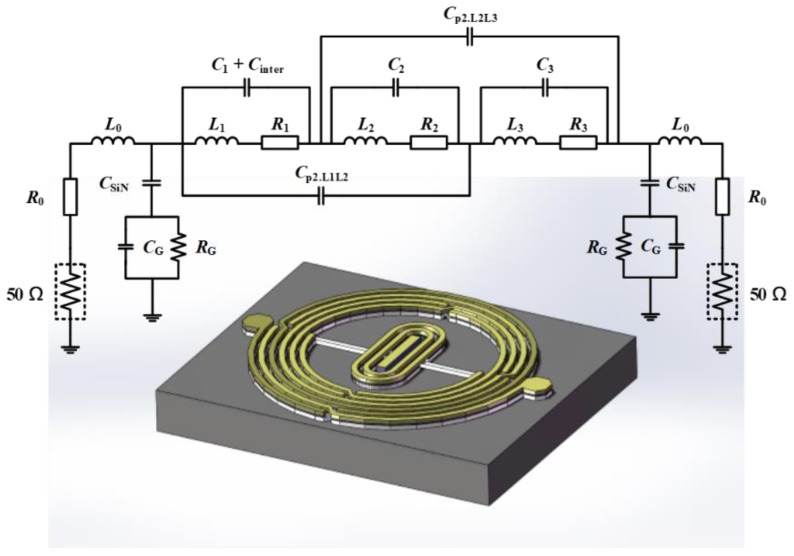
Equivalent circuit of the proposed BPF.

Figure 3 illustrates the complex equivalent circuit model with three laminated layers; *R*_0_ and *L*_0_ represent the resistance and inductance, respectively, of the electrodes and terminations. Because of parasitic effects, the capacitance and resistance of the substrate between the planar pattern and ground take the form of *C*_G_ and *R*_G_, respectively [23]. *R_i_*, *L_i_*, and *C_i_* (*i* = 1, 2, 3) are the resistance, inductance, and coupling capacitance between neighboring turns of the Bond layer, Text layer, and Leads layer, respectively. In addition, *C*_inter_ is the capacitance of the inner quasi-interdigital capacitor of the bottom Bond layer. Moreover, *C*_SiN_ is the capacitance of the SiN_x_ layer between the substrate and the pattern.

The proposed BPF has a multilayer structure, which induces various parasitic effects at high frequencies [24]. The second-order coupling capacitance *C*_p2_ represents the parasitic effect between two adjacent layers and was implemented at high frequencies. The coupling capacitance between these layers is related to the number of layers, the overlay area, and the relative dielectric constant of the materials.

The above-listed parameters *C*_G_, *R*_G_, *C*_SiN_, *R_i_*, *L_i_*, *C_i_*, and *C*_inter_ were calculated as follows:

(5) $$C_{G}=\frac{1}{2}lWC_{\mathrm{sub}}$$

(6) $$R_{G}=\frac{2}{lWG_{\mathrm{sub}}}$$

(7) $$C_{\mathrm{SiN}}=\frac{1}{2}lW\frac{\varepsilon_{\mathrm{SiN}}}{t_{\mathrm{SiN}}}$$

where $C_{\mathrm{sub}}$ and $G_{\mathrm{sub}}$ are the capacitance and conductance per unit area of the gallium arsenide substrate, and $\varepsilon_{\mathrm{SiN}}$ and $t_{\mathrm{SiN}}$ are the dielectric constant and thickness of the SiN_x_ layer separating the pattern and substrate, respectively.

Then,

(8) $$R_{i}=\frac{\rho l}{W\delta \left(1-e^{t/\delta }\right)}$$

(9) $$L_{i}=0.002l\left(ln\frac{2l}{W+t}+0.50049+\frac{W+t}{3l}\right)$$

(10) $$C_{i}=\frac{1}{L_{i}{\left(2\pi f_{0}\right)}^{2}}$$

(11) $$C_{\mathrm{inter}}=0.10\times \frac{\varepsilon_{r}l^{\prime }}{W^{\prime }}$$

where ρ is the resistance coefficient, l is the total circle length, W is the conductor width, δ is the skin depth, t is the thickness of the metal line, $f_{0}$ is the resonance frequency, $\varepsilon_{r}$ is the dielectric constant, $l^{\prime }$ is the finger length, and $W^{\prime }$ is the finger width [25].

### 2.2. Current Density

**Figure 4:**
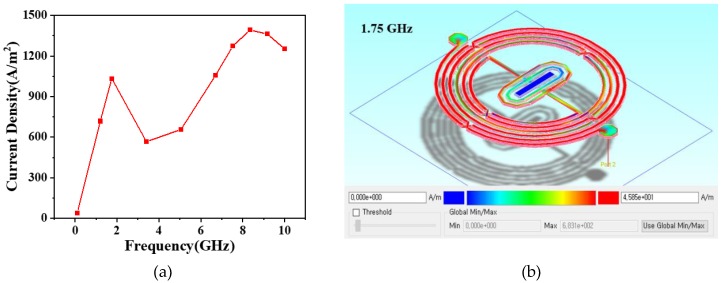
Simulated current density. (**a**) Frequency–current density illustration, and (**b**) Effects of 1.75 GHz on the simulated current density throughout the proposed BPF.

Advanced Design System (ADS) software (Version 2016.01, Keysight Technologies, Inc., Santa Rosa, CA, USA) was employed as a unified interface to simulate momentum in an electromagnetic simulation purpose to evaluate the system design in terms of S-parameter calculation accuracy, surface current, and fields of various planar circuits, including microstrip, stripline, coplanar waveguide, slotline, and other topologies. Interlayer topologies such as air-bridges and vias were incorporated to enable the simulation of multilayer RF/microwave-based printed circuit boards (PCBs), integrated circuits, and multichip modules. Additionally, the simulation was performed in 3D in order to yield a more comprehensive representation of the current flow through slots and conductors through varying degrees of shading, arrows, and contours. The simulated frequency points were selected adaptively for the computation performed through a wide frequency domain that will require a large amount of CPU hardware resources and disk space. Thus, although the frequency range for the electromagnetic (EM) simulation setup was set as 0.1 GHz to 10 GHz, the following 10 frequency points were selected and implemented in the simulation: 0.1 GHz, 1.2 GHz, 1.75 GHz, 3.4 GHz, 5.05 GHz, 6.7 GHz, 7.525 GHz, 8.35 GHz, 9.175 GHz, and 10 GHz. Figure 4a shows the variation of current density under different frequencies with the effects of 1.75 GHz on the simulated current density throughout the proposed BPF illustrated in Figure 4b, which shows that the current flowed through most segments of the simulated BPF with a relatively high density when the frequency was 1.75 GHz, which is within the passband.

## 3. Results and Discussion

**Figure 5:**
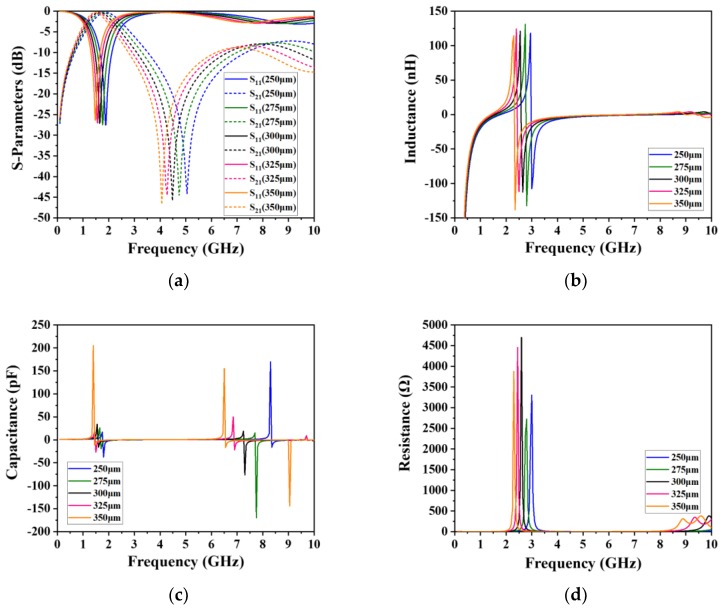
Simulated results illustrating the influence of the inner radius on the respective relationships between S-parameter and lumped parameters and frequency. (**a**) Simulated S-parameters, (**b**) simulated inductance, (**c**) simulated capacitance, and (**d**) simulated resistance.

The influence of the inner radius of the outer inductor on performance was experimentally explored, as this parameter was varied from 250 μm to 350 μm in intervals of 25 μm. Figure 5 illustrates the variation of S-parameter and other lumped parameters as functions of BPF frequency and inner radius. Figure 5a indicates that the positions of the resonant mode and transmission zero tended to shift to areas of lower frequency in response to a larger inner radius. Because the inductance will increase with a bigger inner radius, thus, according to Equation (1), the bigger the inductance, the smaller the resonant frequency. It should also be noted that the impact on S_21_ is more prominent. According to Equations (2)–(4), there are many terms that need to be calculated; we therefore calculated them and tabulated them all in Table 4, which shows that the location of transmission zero definitely moves to areas of lower frequency with the increase of inner radius. Furthermore, inductance, capacitance, and resistance were derived based on the Y-parameters by using the following equations:

(12) $$L\left(\mathrm{nH}\right)=\frac{1.0\times 10^{-9}\times imag\left(\frac{1}{Y\left(1,1\right)}\right)}{2\pi f}$$

(13) $$C\left(\mathrm{pF}\right)=-\frac{1.0\times 10^{-12}}{2\pi f\times imag\left(\frac{1}{Y\left(1,1\right)}\right)}$$

(14) $$R\left(\Omega \right)=real\left(\frac{1}{Y\left(1,1\right)}\right)$$

where *Y* is the admittance, *f* is the frequency of operation, and *imag* and *real* represent the imaginary and real parts, respectively. As illustrated in Figure 5b–d, the first self-resonant frequency tended to decrease as the inner radius was increased. Furthermore, it was confirmed that, at high frequencies, the substrate capacitance reduced the self-resonant frequency (Figure 5c).

**Figure 6:**
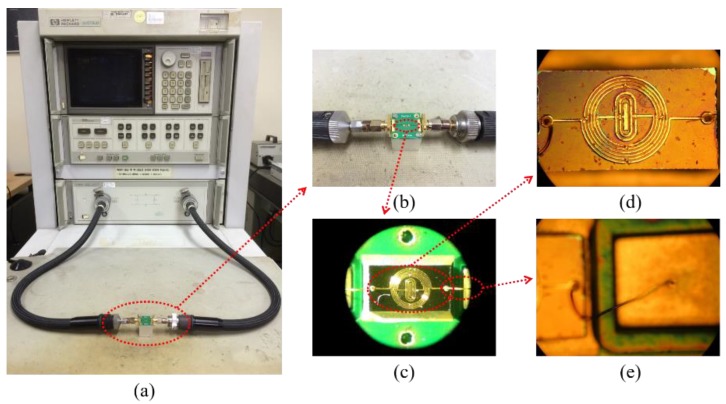
S-parameter measurement setup for the proposed BPF. (**a**) Bandpass filter-vector network analyzer (VNA) cable connection; (**b**) a magnified view of the BPF and its input/output ports; (**c**) 50×-magnified top view of the BPF wire bonded to the printed circuit board (PCB); (**d**) a microscopic view of the fabricated onboard chip; (**e**) a microscopic view showing the BPF input-/output-port wire bond to the input/output pad.

**Figure 7:**
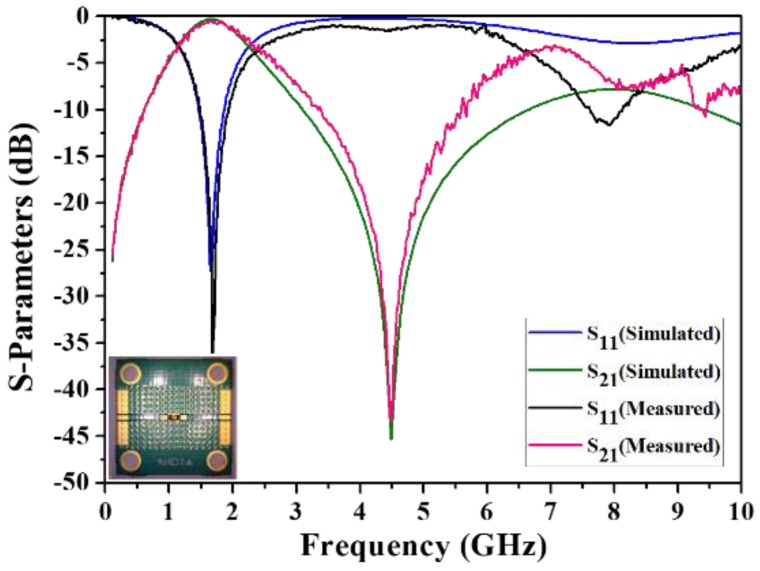
Simulated and measured results for the proposed BPF.

As previously mentioned, the proposed BPF was designed and simulated by using ADS software. In the design of the experimental BPF, the input and output ports were both connected to 50-Ω impedance-matching transmission lines of the PCB by golden wire bonding; the results were subsequently measured and recorded by using an Agilent 8510C vector network analyzer (VNA) (Agilent Technologies, Santa Clara, CA, USA). The experimental setup for the proposed BPF is illustrated in Figure 6 with enlarged views of various parts. The simulated and measured results for the BPF are depicted in Figure 7 along with photographs of the fabricated onboard chip. The results demonstrate that the proposed IPD BPF possesses a single passband with a central frequency of 1.71 GHz (return loss: 32.1 dB, insertion loss: 0.50 dB), and a wide *FBW* of 66.63%. One transmission zero with an amplitude of 43.42 dB was obtained on the right side of the passband at 4.48 GHz. The results of a comparison between the proposed IPD BPF and four previously developed IPD BPFs, as tabulated in Table 5, show that our device features a relatively small chip area and wide *FBW*.

## 4. Conclusions

In this paper, we reported on the design of an IPD technology-based BPF that was fabricated onto a gallium arsenide substrate, and was able to realize a small footprint, a wide *FBW*, a good out-band suppression, and high-frequency operational capability. The chip was fabricated onto a 6-in. gallium arsenide substrate with a dielectric constant of 12.85, loss tangent of 0.006, and thickness of 200.1 µm. Additionally, an equivalent circuit that considers the second-order parasitic effect was modeled in order to simulate device behavior under high-frequency operation. Furthermore, the current distributions were simulated for four different frequencies by using the 3D EM current simulator in ADS software. The influence of the geometric structure was also explored by varying the inner radius. The theoretical predictions and measurements performed on the fabricated bandpass filter were found to be in agreement, thus indicating that the proposed IPD BPF is a good candidate for RF/Microwave applications.

## Figures and Tables

**Table 1:** Dimensional parameters of the Bond layer.

Parameter	*a*	*b*	*c*	*d*	*e*	*f*	*r_in_*
**Unit (μm)**	20	15	1178.5	970	60	258.7	300

**Table 2:** Dimensional parameters of the Text layer.

Parameter	*a*	*b*	*c*	*d*	*e*	*f*	*r_in_*
**Unit (μm)**	16	19	1174.5	966	64	254.7	300

**Table 3:** Dimensional parameters of the Leads layer.

Parameter	*a*	*b*	*c*	*d*	*e*	*f*	*r_in_*
**Unit (μm)**	20	15	1178.5	970	N/A	258.7	300

**Table 4:** Values and calculated results of the terms in Equations (2)–(4).

Terms	$\omega_{\mathrm{mid}}$ (GHz)	$\Omega_{c}$ (GHz)	$\omega_{1}$ (GHz)	$\omega_{2}$ (GHz)	$\omega_{0}$ (GHz)	*FBW* (%)	$w_{0}$ (GHz)
250 μm	2.026	2.749	1.303	2.749	1.893	76.401	5.066
275 μm	1.929	2.625	1.234	2.625	1.800	77.233	4.703
300 μm	1.834	2.519	1.148	2.519	1.701	80.581	4.474
325 μm	1.775	2.451	1.099	2.451	1.641	82.397	4.315
350 μm	1.694	2.352	1.037	2.352	1.562	84.232	4.063

**Table 5:** Comparison of IPD BPF performance.

Reference	Fabrication Process	Circuit Area (mm^2^)	Passband (GHz)	Fractional Bandwidth (%)
[26]	Si-IPD	3.74	2.20	73.00 (3-dB)
[27]	Glass-IPD	<1.00	2.60	49.62 (3-dB)
[28]	Si-IPD	11.6	0.9/2.6	N/A
[29]	Si-IPD	3.9	1.7	16 (10-dB)
This work	GaAs-IPD	1.14	1.71	66.63 (3-dB)
